# User perceptions of individually-tailored health information in digital apps: development of a scale

**DOI:** 10.3389/fdgth.2026.1731948

**Published:** 2026-02-25

**Authors:** Raymond L. Ownby, Rosemary Davenport, Joshua Caballero

**Affiliations:** 1 Center for Brain Health and Longevity, Department of Psychiatry and Behavioral Medicine, Nova Southeastern University, Fort Lauderdale, FL, United States; 2 Department of Clinical and Administrative Pharmacy, University of Georgia, Athens, GA, United States

**Keywords:** activation, chronic disease, health information, health literacy, mobile app, self-efficacy, self-management, tailoring

## Abstract

**Objectives:**

Individually tailored health information is thought to have greater effects on patient behavior than generic advice because it is more personally relevant. Most digital health studies, however, do not actually measure the effect of tailoring on study outcomes. To address this gap, we created the Success in Tailoring (SIT) scale which assesses how users perceive information as relevant, useful, and actionable.

**Methods:**

The SIT items were chosen to reflect theoretical work on relevance and elements of the Elaboration Likelihood Model. It was administered to participants in a study of a mobile app providing tailored information about chronic disease self-management to persons 40 years of age and older with low health literacy. Participants responded immediately after completing the study intervention and again three months later. Psychometric analyses focused on the measure's reliability, factor structure, and convergent and divergent validity other measures thought to be related and unrelated to it. We assessed test-retest reliability and factorial invariance over administration, and whether the measure predicted changes in key study outcomes.

**Results:**

Analyses were based on responses from 275 participants. The SIT's internal consistency was good, and test-retest reliability was acceptable. Exploratory factor analysis suggested a single-factor solution, although subsequent confirmatory analyses revealed that a bifactor solution with a robust general factor and two minor subfactors fit the data best. The scale was significantly correlated with measures related to its underlying concept and unrelated to measures not related to it, such as physical and cognitive status. Configural and metric, but not scalar, factorial invariance, were confirmed. SIT scores were related to change in activation and disease-management self-efficacy over the course of the study. Confirmatory bifactor analyses supported treating the SIT as essentially unidimensional, with a single total score providing a reliable and valid index of perceived tailoring.

**Conclusion:**

The SIT gives researchers a straightforward means of capturing whether participants feel information is successfully tailored to them. It may be helpful in explaining how personalization, a key feature of many digital health apps, may be related to outcomes.

## 1 Introduction

Tailored health communication has long been recognized as more effective than generic information in promoting patient engagement and behavior change (1). Health communications that take account of a person's own interests and needs are perceived as more relevant, processed more deeply, and are more likely to produce behavior change (2, 3). In the context of digital health interventions, tailoring is particularly important, as apps and online tools can adapt content dynamically to user responses and behaviors (2).

While extensive research supports the usefulness of tailoring (1, 4), few studies have quantified it when implemented. The theoretical effectiveness of tailoring is well established, but there is a methodological gap in how researchers currently measure and verify its implementation. Research on digital tools often still points to clicks, logins, or general outcomes and treats them as evidence of effectiveness (5). To address this issue, we created the Success in Tailoring (SIT) scale, a brief measure of how well participants feel intervention content fits their needs and circumstances. The scale grew out of the Elaboration Likelihood Model (ELM) and was informed by qualitative work with adults managing multiple chronic conditions (6). Items were chosen to reflect an underlying model of the dimensions of information relevance (7, 8), including the extent to which a person viewed the tailored information as related to their situation, their needs, and the extent to which it was useful.

The SIT scale was used in a study of a tailored information app for chronic disease self-management (9), and we showed, for example, that users' SIT scores were related to change in stress, which was in turn related to improved quality of life (10). The SIT has promise, but it is still missing a more extensive formal validation. Testing its reliability, factor structure, and links to related measures like patient activation, self-efficacy, and health-related quality of life would give the scale a stronger foundation. The purpose of this paper is to report this more extensive analysis of the reliability and validity of the SIT scale.

## 2 Methods

### 2.1 Parent study

#### 2.1.1 Participants and procedures

In the study for which the SIT was developed, participants were adults aged 40 and older with at least one chronic health condition and low health literacy. They were recruited from local clinics, previous studies, and community outreach (9). Eligibility required less than a college education and a score below the 8th-grade cutoff on the Rapid Estimate of Adult Literacy in Medicine or REALM (11). Participants represented a wide range of chronic conditions, including both physical and mental health diagnoses, and all were receiving treatment with at least one prescribed or recommended medication.

The parent study evaluated a chronic disease self-management (CDSM) app designed for individuals with low health literacy. The app featured three versions, each presenting the health content at different reading levels (8th, 6th, and 3rd grade, the latter with audio narration). Content was developed by a multidisciplinary team and included extensive qualitative interviewing of relevant stakeholders (6) and was refined through multiple rounds of usability testing with the target population. Participants completed an extensive baseline assessment—including demographic information, medical and educational history, and standardized measures of reading ability and health literacy—either individually-administered or via audio computer-assisted self-interview software to minimize literacy-related response bias.

Health information related to chronic disease self-management of common problems related to multimorbidity was provided in a series of modules focused on topics such as treatment adherence, sleep, mood, fatigue, and pain. Each module consisted of a series of screens providing information on the topic. Individual tailoring was accomplished at the beginning of each module by asking participants to respond to questions related to difficulties in each area. For example, in the sleep module participants completed questions that asked about problems falling or staying asleep or waking up too early. They also responded to queries about how refreshing their sleep was and how sleep problems affected their daily activities. Later in the module, participants were provided with information related to the difficulties they reported, such as relaxation techniques or mediation for difficulties falling asleep.

For outcome assessment, widely used and validated instruments were administered at baseline, immediately following the intervention, and three months post-intervention. These included measures of patient activation (12), self-efficacy (13), health-related quality of life (14), and medication adherence (15). The measure under validation in the present study was embedded within this comprehensive assessment protocol. A total of 275 participants responded to the SIT immediately after completing the study intervention, and 240 completed it at the three-month follow-up.

#### 2.1.2 Item development

We developed the SIT items with the idea that people use different cues when they decide whether health information feels important. Saracevic's reviews of relevance were our starting point, and we also kept the Elaboration Likelihood Model in mind as a way of thinking about personal relevance. Items were generated by the first author (RLO) after consideration of theoretical sources, including relevance and the elaboration likelihood model. Limitations in available staff and resources and the fact that the scale was a small part of a much larger project that was an intervention study (9) precluded more extensive item development through interviews and cognitive testing. We assessed the readability of items using Health Literacy Advisor (Bethesda, MD, USA: Health Literacy Innovations, LLC) to ensure that participants would be able to read them, although the measure was administered by software that read them aloud to participants (Bethesda, MD, USA: Questionnaire Development System). See Table 1 for links between items, dimensions of relevance, and the ELM.

**Table 1:** Success in tailoring (SIT) items mapped to relevance frameworks and the elaboration likelihood model.

SIT item	Manifestation(s) of Relevance	Clues/Criteria	Stratified Model Layer	ELM Link
I could relate to the information.	Situational, socio-cognitive	Use/situational match; cognitive match	Cognitive, affective, situational	Personal relevance → central route processing
The information was about people like me.	Socio-cognitive	Use/situational; belief match (identity/credence)	Socio-cognitive	Self-relevance cue; identification with message
The information would be useful to someone like me.	Situational	Use/situational match; topical	Situational	Relevance to personal goals enhances elaboration
The information was about the kind of problems people like me have.	Topical + socio-cognitive	Content; use/situational	Cognitive + situational	Topic + identity match increases elaboration likelihood
The information was easy to understand.	Cognitive	Cognitive match (understanding/effort)	Cognitive	Comprehension facilitates elaboration
The information would work for me.	Situational/utility	Use/situational (utility)	Situational	Perceived applicability strengthens motivation to act
The information gave me something to think about.	Cognitive + affective	Cognitive match (novelty); affective match (engagement)	Cognitive + affective	Message elaboration (deep thought)
I want to try out some of the things talked about.	Affective/motivational	Affective match; use/situational (intended use)	Affective, situational	Intention → behavior (ELM outcome of central route)

Some of the items are clearly direct, like asking whether the material was “about people like me” or “about the kind of problems people like me have.” That was meant to tap the social and topical aspect of health information—whether people see themselves reflected in the content. Others focus more on the practical side, such as whether the information seemed useful or whether it “would work” for them. We also added an item about whether it was “easy to understand,” a basic check on comprehension or cognitive fit. Finally, several items go further, for instance, “gave me something to think about” or “I want to try out some of the things.” Those are intended to assess a participant's motivation and affect—did the material generate interest or a plan to act?

The SIT scale maps the transition from initial perception to the “rationalization” or elaboration of the health message. According to the ELM, when a user identifies information as “about people like me” (social relevance) and find it “easy to understand” (cognitive relevance), they are more likely to process the information through the central route (16). This leads to the item “gave me something to think about,” which directly captures the elaboration or rationalization of the tailored content. We hypothesize that this process is what ultimately drives changes in patient activation and self-efficacy.

Overall, then, the items assess the main aspects of relevance—cognitive, practical, and affective—with the kinds of clues people usually use to judge information. In relation to the Elaboration Likelihood Model, the items follow its elements. Starting with personal relevance and comprehension, moving through elaboration (“gave me something to think about”), and ending with intention (“I want to try out…”). The scale is therefore tied to both relevance theory and mechanisms thought to connect tailoring with changes in health behavior.

### 2.2 Psychometric evaluation

#### 2.2.1 Measures

##### 2.2.1.1 Success in tailoring (SIT)

The SIT was designed as a brief measure of participants' perceptions of health information as personally relevant. Participants were asked to rate each item on a five-point Likert-type scale, and responses were summed to create a total score. Higher scores indicate greater perceived individual tailoring of the information.

##### 2.2.1.2 External measures

To evaluate validity, we included several established constructs. The Patient Activation Measure (PAM) (12) and the Chronic Disease Self-Efficacy Scale (CDSE) (13) were used as convergent measures, as both assess constructs expected to relate closely to tailoring perceptions. Divergent validity was tested using scales less directly tied to tailoring, such as cognitive and academic measures and physical assessments. Predictive validity was assessed by evaluating the SIT's ability to predict change in outcome measures over time.

#### 2.2.2 Procedure

The SIT was administered at two points: immediately after participants completed the intervention and again three months later. Cognitive and academic measures were administered only at baseline, and physical status (height, weight, blood pressure, walking speed) was also only assessed at baseline. Ethical approval for the study was obtained from the Institutional Review Boards of Nova Southeastern and Emory Universities. Participants gave verbal consent for screening, and all participants gave written informed consent for further participation.

#### 2.2.3 Analytic strategy

Descriptive statistics were computed in SPSS versions 29 and 30. Most other analyses were completed using packages in R (version 4.3.1)**.** Mplus version 9 was used to create latent growth curve models. To evaluate the dimensionality of the SIT, we estimated a confirmatory bifactor model in which all items loaded on a general perceived-tailoring factor as well as on one of several content-specific group factors representing different aspects of tailored communication. Factors were specified as orthogonal. Model fit was compared with one- and two-factor solutions using robust maximum likelihood estimation (MLR) in *lavaan (*17). To assess the relative strength of the general vs. group factors, we calculated bifactor indices including the Explained Common Variance (ECV), Percent Uncontaminated Correlations (PUC), Omega Total (*Ω*_T_), Omega Hierarchical for the general (*Ω*_H_ and group factors (*Ω*_HS_), construct replicability (H), and factor determinacy (FD) using the *BifactorIndicesCalculator (*18) and *semTools (*19) packages in R. Thresholds recommended in the literature (e.g., ECV ≥ 0.60, PUC ≥ 0.70, *Ω*_H_ ≥ 0.70) were used to evaluate whether the measure could be considered essentially unidimensional (20, 21). Together, these indices provided a comprehensive picture of the SIT's dimensional structure and the degree to which a single score could represent the underlying construct of perceived tailoring. Issues related to missing data were addressed by using Full Information Maximum Likelihood (FIML) estimation in all analyses as recommended by Enders (22).

##### 2.2.3.1 Exploratory factor analysis (EFA)

An exploratory factor analysis was used to examine the structure of the SIT items. Analyses relied on the *psych (*23) and *nFactors* (24) packages. The number of factors was guided by parallel analysis (25). Principal axis factoring was used with evaluation of both varimax and oblimin rotations.

##### 2.2.3.2 Confirmatory factor analysis (CFA)

Competing models (one-factor, two-factor, and bifactor) were tested in *lavaan* (17). Fit was evaluated using multiple indices: the Comparative Fit Index (CFI), Tucker–Lewis Index (TLI), Root Mean Square Error of Approximation (RMSEA) with 90% confidence interval, and the Standardized Root Mean Square Residual (SRMR). Because item distributions were moderately skewed, all latent variable analyses were conducted using robust maximum likelihood estimation (MLR), which provides standard errors and *χ*^2^ statistics robust to non-normality.

##### 2.2.3.3 Longitudinal measurement invariance

Measurement invariance across the two administrations was evaluated in *lavaan*. Models were estimated using robust maximum likelihood (MLR) in *lavaan*, which provides standard errors and fit indices corrected for non-normality (Satorra–Bentler scaled) (26). Configural, metric, and scalar models were estimated, and changes in fit indices were used as criteria for invariance (*Δ* CFI ≤ .01, *Δ* RMSEA ≤ .015 (27, 28) as well as change in the likelihood ratio chi-square. Robust chi-square difference tests (via *lavTestLRT* from the *lavaan* package) were used to compare nested models, using the Satorra–Bentler correction for MLR estimation.

##### 2.2.3.4 Reliability analyses

Test–retest reliability was estimated with intraclass correlations (ICCs) using reliability analysis in SPSS 29. Both absolute agreement [ICC(A,1)] and consistency [ICC(C,1)] were reported with 95% confidence intervals. Precision was further described with the standard error of measurement (SEM) and the smallest detectable change (SDC) at the 95% level. Agreement at the individual level was assessed with Bland–Altman plots (29) produced in *ggplot2* (30), which showed mean bias, 95% limits of agreement, and a regression line to test for proportional bias. We also produced a spaghetti plot of SIT scores at the two time points to visually assess change over time.

##### 2.2.3.5 Convergent and divergent validity

Convergent validity was examined by correlating SIT scores with constructs expected to be related, including perceived usefulness of the app (Usefulness scale of a Technology Acceptance Model-based user feedback scale) (31); patient activation (Patient Activation Measure, PAM) (12); participant level of health literacy (Test of Functional Health Literacy in Adults, TOFHLA, and FLIGHT/VIDAS Health Literacy Measure, FVA) (32, 33); internal locus of control (Internal Control subscale of the Multidimensional Health Locus of Control Scale, MHLC) (34); disease management self-efficacy (Chronic Disease Self-Efficacy Scale, CDSE) (13). Divergent validity was examined as correlations of constructs expected to be unrelated, including participant self-report of annual income; stress (Perceived Stress Scale, PSS) (35); depressive symptoms (Center for Epidemiological Studies—Depression scale, CESD) (36); participant report of total number of chronic health conditions; age in years; participant systolic blood pressure before completing the 10-meter walk test and time taken in the 10-meter walk test (37). Pearson correlations were computed with base R functions.

##### 2.2.3.6 Predictive validity

Predictive validity was tested using latent growth models in Mplus 9 (38). Latent growth curve models, including the parent study's primary outcomes of patient activation and disease management self-efficacy, were constructed in several stages. In the first stage, growth curves were calculated to assess whether the variables had changed over the course of the study by testing whether the slope was significantly different from zero. For each growth curve, its intercept and slope were then regressed on potentially important covariates, age, gender, and race to account for potential confounding variables. Finally, the slopes were regressed on the SIT score to determine whether SIT scores were related to change in outcomes over the course of the study. Health-related quality of life at the three-month follow-up (QOL; SF36 General Health scale) (14) was then regressed on the slopes, and the indirect effect of the SIT on QOL was evaluated.

## 3 Results

### 3.1 Sample characteristics

Descriptive information for the sample is presented in Table 2. The sample included 275 participants aged 40 years and older with low health literacy and at least one chronic health condition for which they were being treated. The most common chronic conditions they reported were elevated cholesterol levels, high blood pressure, and type II diabetes. The sample included 147 women (53.5%) 37 Whites, and 238 Black, Indigenous, and other People of Color individuals. Most had few socioeconomic resources, with more than half reporting annual incomes of less than $15,000 per year. Participants' digital literacy was assessed with the eHealth Literacy Scale (39). Interpretation of our findings on this measure is complicated by the lack of formal norms for it, but one review reported finding a weighted mean score drawn from 20 publications of 24.3 (95%CI 17.1–31.6) (40), suggesting that our participants were similar to those in other studies.

**Table 2:** Descriptive statistics for the sample (*n* = 275).

Variable	Minimum	Maximum	Mean	SD
Age in years	40	90	57.24	8.21
Education in years	4	15	11.81	1.89
Total Health Conditions	1	18	6.63	2.69
Reading Grade Score	1.2	18.0	7.13	4.04

Total Health Conditions, Total number of health conditions reported by participants. Reading Grade Score, Woodcock-Johnson Psych-Educational Battery Passage Comprehension subtest, reading grade.

### 3.2 Item characteristics

Individual item statistics are presented in Table 3. Item-level descriptive statistics (Table 2) indicated uniformly high mean scores, ranging from 4.19 to 4.32 on a 5-point scale. All items showed moderate to strong negative skew (−1.23 to −1.62) and positive kurtosis (3.09–4.90), suggesting that participants tended to endorse the upper end of the response scale and that responses were tightly clustered. This pattern reflects generally favorable perceptions of information tailoring and limited variability among respondents. This pattern is consistent with the scale's intent and the intervention's design but may constrain the ability to detect associations with other variables due to reduced variability.

**Table 3:** Fit indices for single factor and bifactor confirmatory analyses.

Model	CFI	TLI	RMSEA	SRMR
One Factor	0.92	0.89	0.17	0.05
Bifactor	0.96	0.93	0.14	0.04

CFI, confirmatory fit index; TLI, Tucker–Lewis index; RMSEA, root mean square error of approximation; SRMR, standardized root mean square residual.

Inter-item correlations are presented in Table 4. Correlations among the eight SIT items were all positive, statistically significant (all *p*s < 0.01), and of moderate to strong magnitude (Table 3). Correlations ranged from *r* = 0.51 to 0.83, with a median of approximately.70, indicating substantial coherence among items. The strongest associations were observed between items reflecting the applicability and thought-provoking qualities of the information (“I could use the information” and “Gave me something to think about,” *r* = 0.83). In contrast, the weakest associations involved relational or engagement-oriented items (e.g., “I could relate to the information” and “I want to try out some of the things,” *r* = 0.51). This pattern suggests that while the items assess different aspects of perceived tailoring, they share a core consistent with a single underlying construct.

**Table 4:** Inter-item correlations.

Item	I could relate to the information	About people like me	Easy to understand	I could use the information	Gave me something to think about	About problems people like me have	Would work for me	I want to try out
I could relate to the information	1.00	0.69	0.70	0.54	0.53	0.57	0.57	0.51
About people like me	0.69	1.00	0.74	0.75	0.73	0.72	0.62	0.56
Easy to understand	0.70	0.74	1.00	0.75	0.74	0.68	0.68	0.64
I could use the information	0.54	0.75	0.75	1.00	0.83	0.78	0.75	0.66
Gave me something to think about	0.53	0.73	0.74	0.83	1.00	0.81	0.75	0.70
About problems people like me have	0.57	0.72	0.68	0.78	0.81	1.00	0.75	0.68
Would work for me	0.57	0.62	0.68	0.75	0.75	0.75	1.00	0.78
I want to try out some of the things	0.51	0.56	0.64	0.66	0.70	0.68	0.78	1.00

All correlations are significantly different from zero (all *p*s < 0.01).

### 3.3 Exploratory factor analysis (EFA)

**Figure 1:**
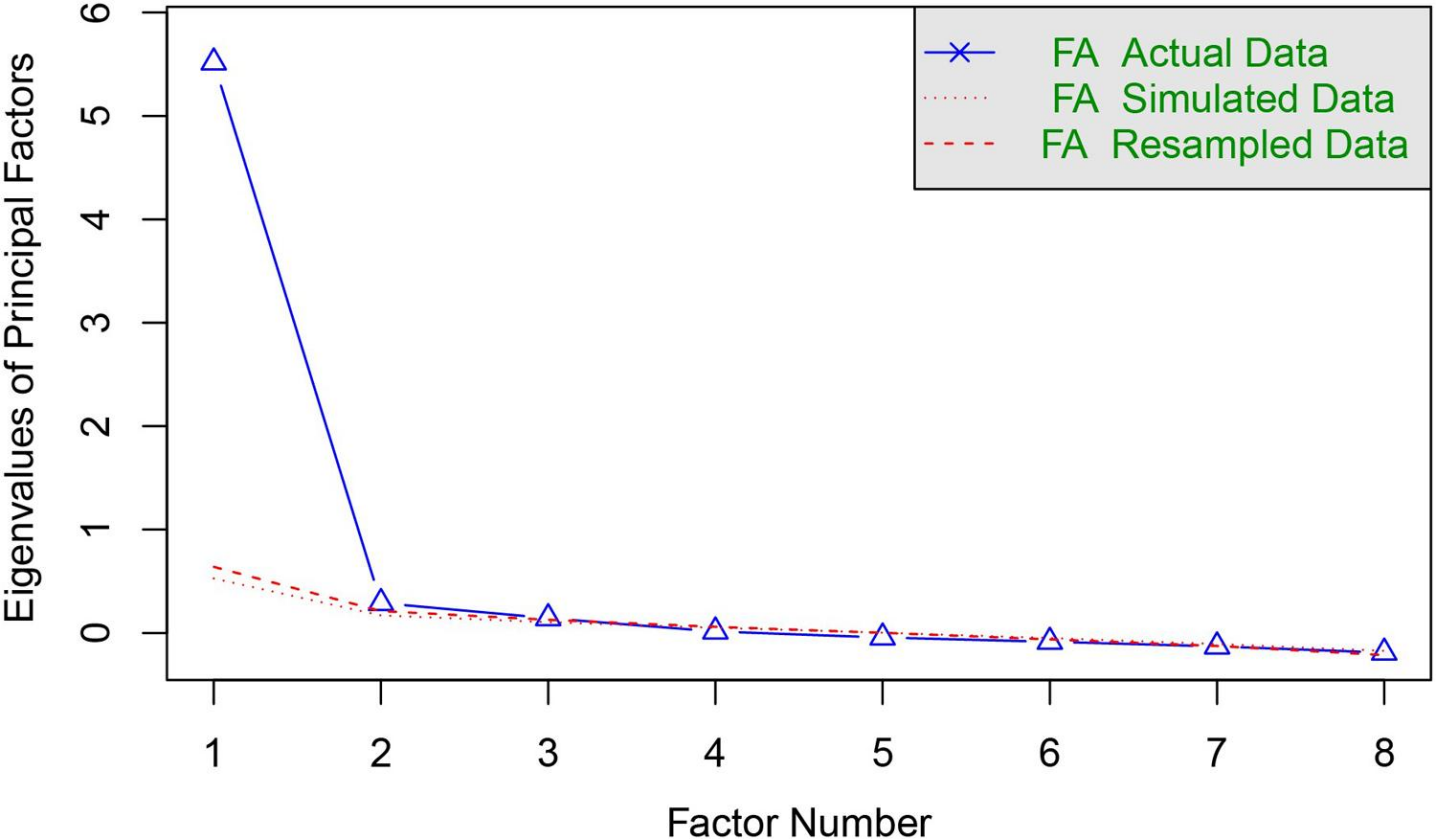
Scree plot of parallel factor analysis. FA, factor analysis.

Exploratory analyses with parallel analysis suggested a single-factor solution (Figure 1). Most SIT items loaded well on the first factor. Table 5 lists the factor loadings for the one-factor solution. Overall, the pattern of loadings is consistent with a unidimensional structure.

**Table 5:** Exploratory factor loadings.

Item	Loading
I could relate to the information	0.67
About people like me	0.82
Easy to understand	0.83
I could use the information	0.90
Gave me something to think about	0.90
About problems people like me have	0.87
Would work for me	0.84
I want to try out some of the things	0.77

### 3.4 Confirmatory factor analysis (CFA)

To further assess the factor structure of the SIT scale, we fit a single-factor confirmatory factor analytic model. It fit the data moderately well (CFI = 0.92, TLI = 0.89, RMSEA = 0.17, SRMR = 0.05) but not at an acceptable level. Considering this evidence of misfit, we explored several alternate factor models; the one that fit the data best was a bifactor model with one general and two minor factors. The first minor factor included the items “The information would work for me” and “I want to try out some of the things talked about,” while the second included “The information was easy to understand” and “I could relate to the information.” Fit indices were substantially improved (CFI = 0.96, TLI = 0.93, RMSEA = 0.14, SRMR = 0.04). The bifactor model fit the data significantly better than the single-factor model [likelihood ratio test *χ*^2^ (4) = 42.22, *p* < 0.001].

The bifactor model thus provided the best overall fit among competing structures. Model-level indices indicated that variance in SIT responses was dominated by a strong general factor representing perceived tailoring. The general factor accounted for 88% of the common variance (ECV = 0.88), with a high proportion of uncontaminated correlations (PUC = 0.93). Reliability coefficients were excellent (*Ω*_T_ = 0.96; *Ω*_H_ = 0.93), indicating that most reliable variance in total SIT scores reflected the general factor. Construct replicability (H = 0.96) and factor determinacy (FD = 0.98) were also high, supporting stable estimation of the general factor. In contrast, the specific factors showed low unique reliability (*Ω*_HS_ = 0.18–0.22) and modest replicability (H = 0.28–0.38), suggesting limited distinct variance beyond the general factor. Together, these results indicate that the SIT functions mainly as a unidimensional measure of perceived tailoring, and that a single total score provides a psychometrically useful representation of the construct.

### 3.5 Longitudinal measurement invariance

Configural, metric, and scalar models were compared for the bifactor model across administrations (Table 6). Fit indices changed only slightly moving from configural to metric invariance and stayed within the cutoffs. The CFI and RMSEA changed minimally from configural to metric models [*Δ* CFI = −.01, *Δ* RMSEA = 0.00; *Δχ*^2^ (8) = 8.31, *p* = 0.40], indicating equivalent factor loadings across assessments. These results supported metric invariance, suggesting that the SIT items operated in a consistent way over time with similar factor loadings. However, fit indices did not support scalar invariance. Constraining item intercepts (scalar model) reduced fit substantially (*Δ* CFI = −.05, *Δ* RMSEA = + .02), consistent with the significant *χ*^2^ difference [*Δχ*^2^ (6) = 131.16, *p* < 0.001]. Scalar invariance was thus not supported.

**Table 6:** Fit indices for factorial invariance models.

Index	Configural	Metric	Scalar
CFI	0.91	0.90	0.85
TLI	0.88	0.89	0.84
RMSEA	0.11	0.11	0.13
SRMR	0.05	0.08	0.22

### 3.6 Reliability analyses

Test–retest reliability across administrations was moderate. The intraclass correlation coefficient (ICC) was calculated as a single measure with a two-way random-effects in both absolute-agreement and consistency models. The ICC for absolute agreement [ICC(A,1)] was 0.60 (95% CI 0.51–0.69), and the consistency ICC [ICC(C,1)] showed a similar pattern (ICC = 0.61, 95% CI 0.52–0.70). The standard error of measurement (SEM) was 3.63, giving a smallest detectable change (SDC) of 10.07. Cronbach's alpha for the scale at time 1 was 0.95; at time 2, it was 0.93. McDonald's omega was 0.95. at time 1 and 0.93 at time 2.

**Figure 2:**
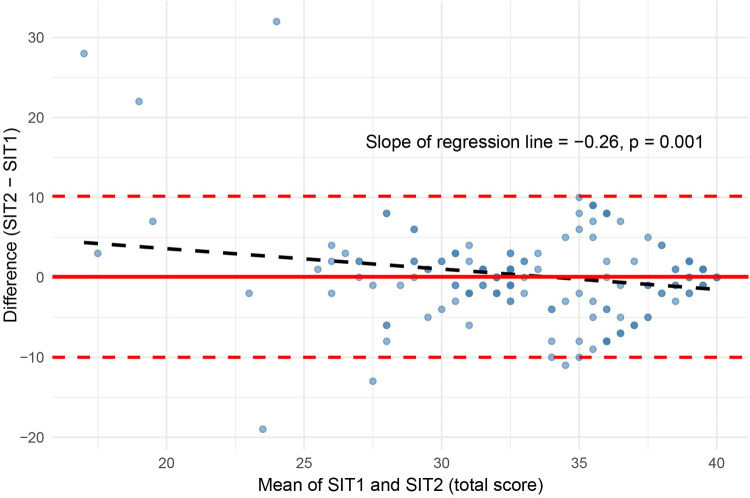
Bland–Altman plot with regression line.

**Figure 3:**
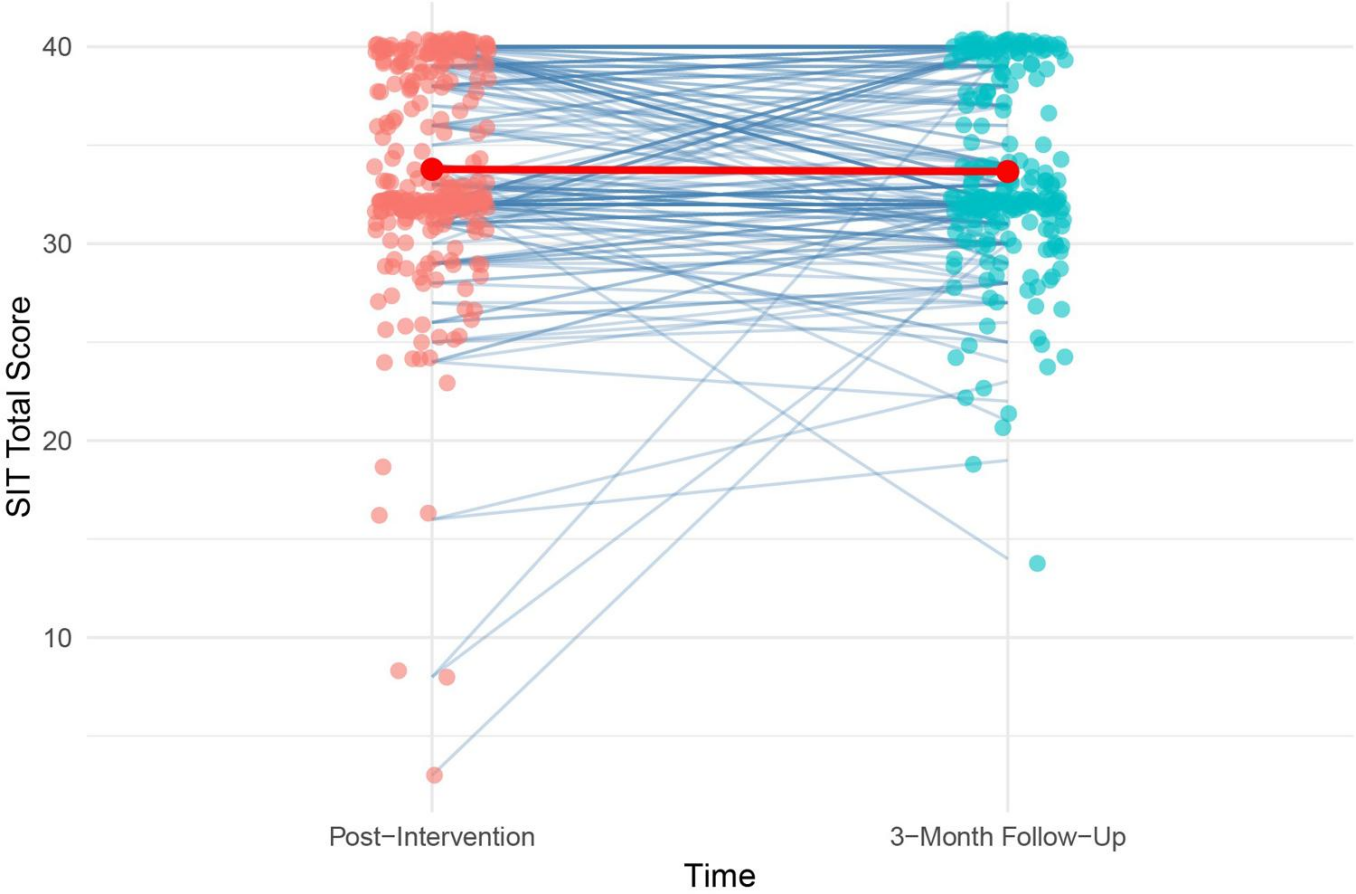
Spaghetti plot of SIT1 vs. SIT2 scores.

A Bland–Altman plot was used to examine agreement between the two SIT administrations Figure 2. The mean difference between scores was close to zero. However, regressing score differences on their means revealed a statistically significant negative slope (*β* = −0.26, *p* = .001), suggesting a proportional bias: participants with lower average SIT scores tended to show slightly higher second-administration scores, whereas those with higher average scores showed smaller or slightly negative differences. Despite this trend, most differences fell within the 95% limits of agreement, indicating good overall test–retest consistency. A spaghetti plot of individual SIT scores (Figure 3) shows variability across participants in the two administrations, even though the group means remained stable (red line in Figure 3).

### 3.7 Convergent and divergent validity

**Figure 4:**
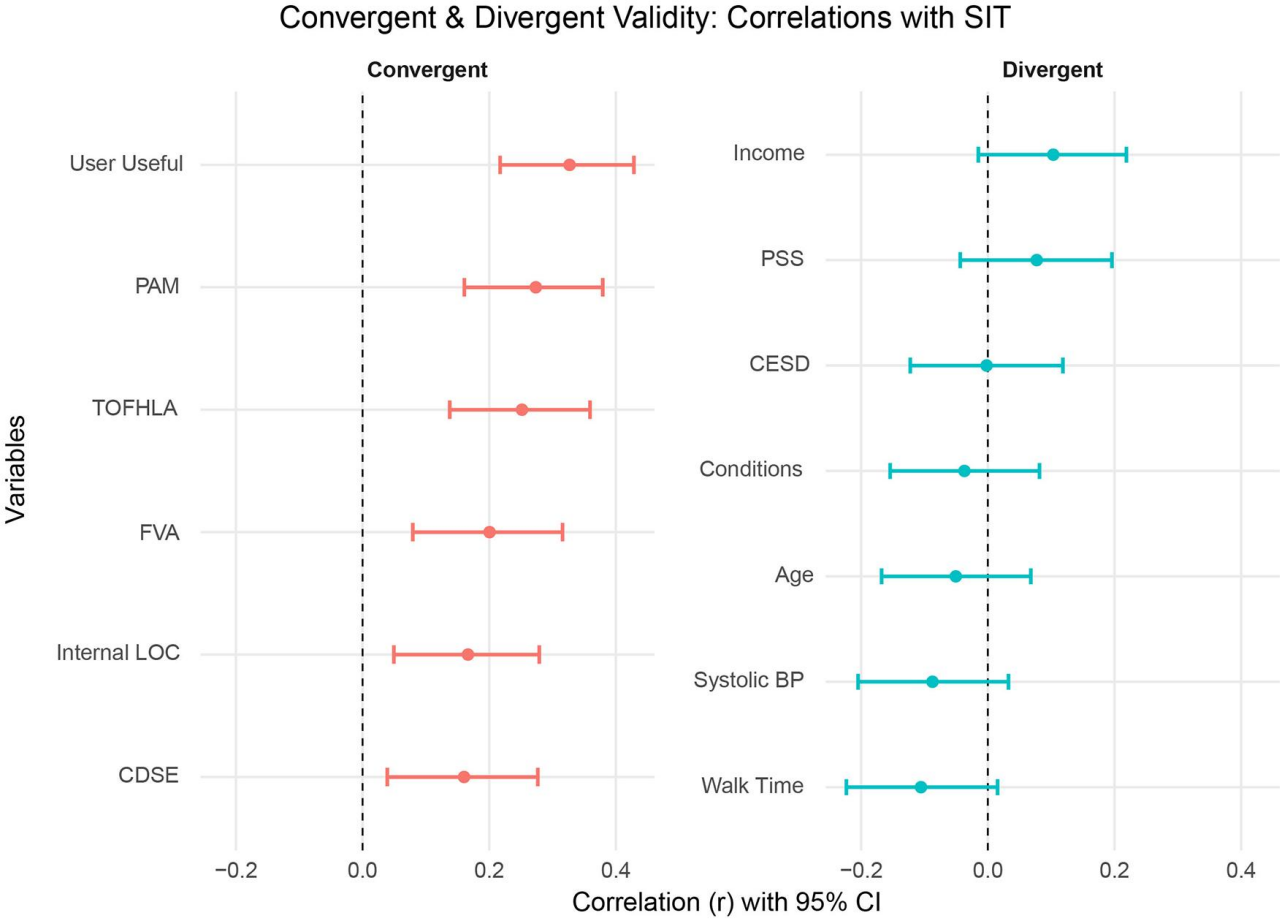
Correlations of SIT with convergent and divergent measures*.* User Useful, Usefulness scale of Technology Acceptance Model scale; PAM, Patient Activation Measure; TOFHLA, Test of Functional Health Literacy in Adults; FVA, FLIGHT/VIDAS Health Literacy Measure; Internal LOC, Internal control subscale of the Multidimensional Health Locus of Control Scale; CDSE, Chronic Disease Self-Efficacy Scale; Income, Participant self-report of income; PSS, Perceived Stress Scale; CESD, Center for Epidemiological Studies—Depression scale; Conditions, Participant report of total number of chronic health conditions; Age, age in years; Systolic BP, Participant systolic blood pressure before completing the 10-meter walk test; Walk Time, Time taken in the 10-meter walk test.

As expected, SIT scores correlated positively with measures assessing related constructs (Figure 4). Perceived tailoring was most strongly associated with participants' ratings of the information's usefulness (User Useful; *r* = .33, *p* < .001) and with patient activation (PAM; *r* = .27, *p* < .001). Smaller but still significant correlations were observed with health literacy (TOFHLA; *r* = .25, *p* < .001; FVA; *r* = .20, *p* = .001), internal health locus of control (*r* = .17, *p* = .006), and chronic disease self-efficacy (*r* = .16, *p* = .010). In contrast, SIT scores were not significantly related to demographic and health variables—including income, age, number of chronic conditions, and systolic blood pressure—and to psychological measures of perceived stress (PSS; *r* = .08, ns) and depressive symptoms (CESD; *r* = 0.002, ns). This pattern demonstrates good convergent validity with conceptually related psychosocial constructs and divergent validity with unrelated health and demographic factors.

### 3.8 Predictive validity

**Figure 5:**
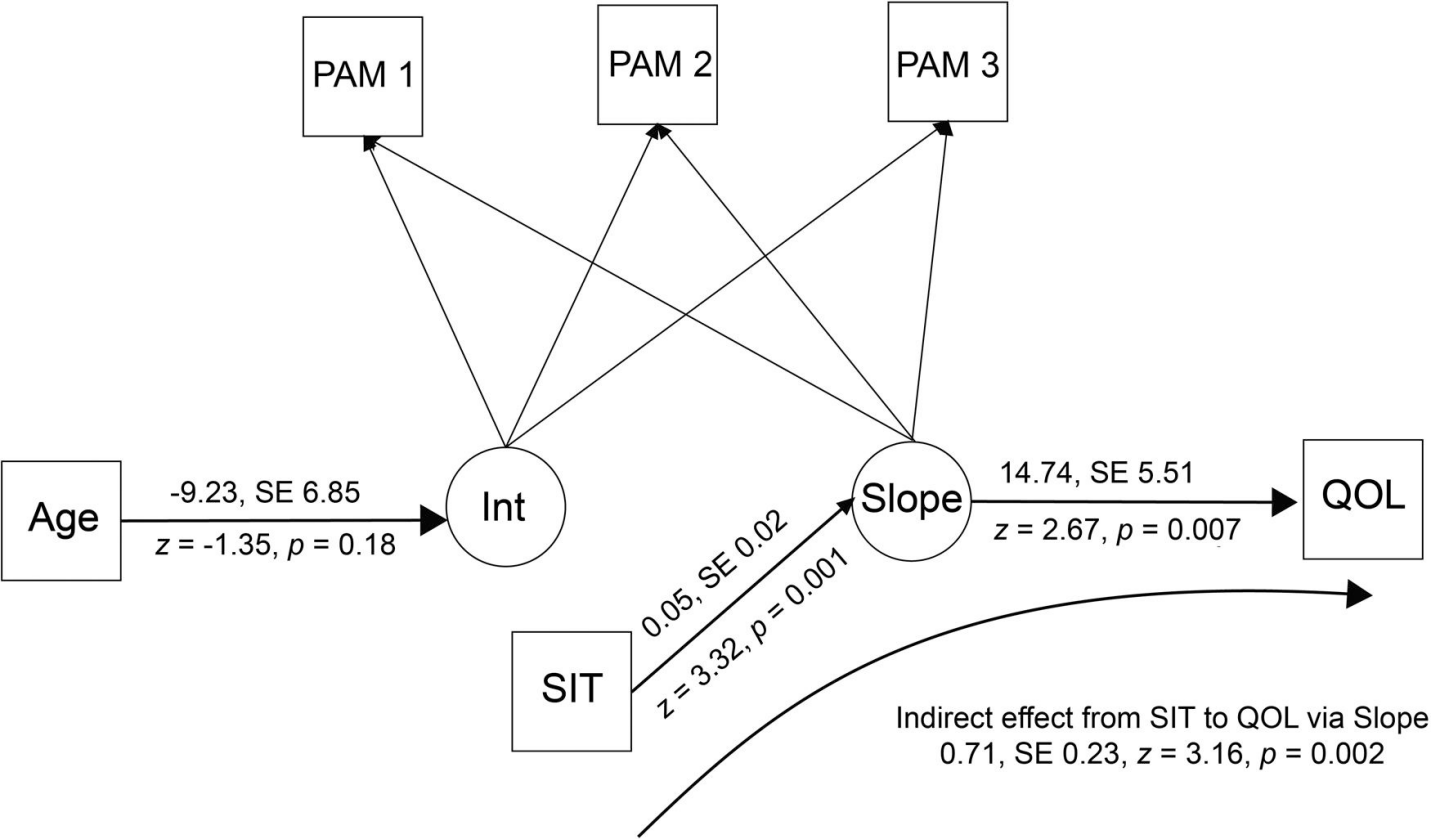
Latent growth curve model for patient activation*.* PAM, Patient Activation Measure; Age, age in years; Int, model intercept; Slope, model slope; QOL, SF36 General Health scale at three-month follow-up; SIT, Success in Tailoring scale score.

The latent growth models incorporating patient activation (PAM) and chronic disease self-efficacy (CDSE) showed that SIT scores obtained immediately after the intervention predicted change in outcomes over time. Figure 5 shows the growth curve for patient activation. Patient activation changed significantly over the course of the study (coefficient = 0.17, SE = 0.08, *z* = 2.08, *p* = 0.04). The regression of the model's slope on the SIT score was also significant (coefficient = 0.05, SE = 0.02, *z* = 3.32, *p* = 0.001) as was the regression of the QOL measure (SF36 General Health scale at three-month follow-up (coefficient = 14.73, SE = 5.51, *z* = 2.67, *p* = 0.007). The indirect effect of the SIT on QOL via change in activation was also statistically significant (estimate = 0.71, SE = 0.23, *z* = 3.16, *p* = 0.002).

**Figure 6:**
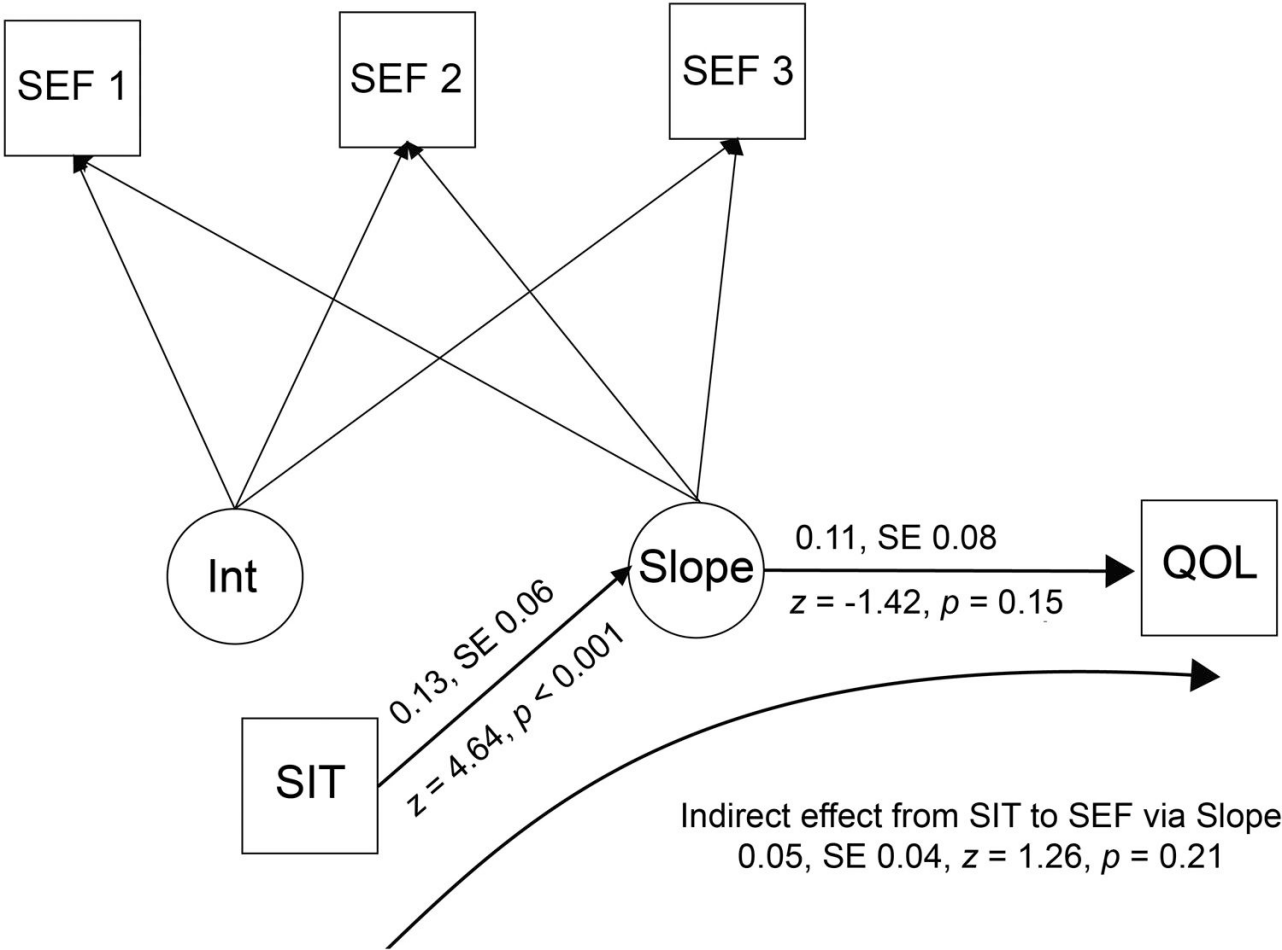
Latent growth curve model for self-efficacy*.* SEF, Chronic Disease Self-Efficacy Scale; Int, model intercept; Slope, model slope; QOL, SF36 General Health scale at three-month follow-up; SIT, Success in Tailoring scale score.

Figure 6 shows the growth curve for chronic disease self-efficacy. It increased significantly over the course of the study (coefficient = 1.26, SE = 0.27, *z* = 4.64, *p* < 0.001). The regression of the model's slope on the SIT score was also significant (coefficient = 0.13, SE = 0.06, *z* = 2.26, *p* = 0.02), but the regression of the QOL measure (SF36 General Health scale at three-month follow-up; (coefficient = −0.11, SE = 0.08, *z* = −1.42, *p* = 0.15) was not. The indirect effect of the SIT on QOL was not statistically significant (estimate = 0.05, SE = 0.04, *z* = 1.26, *p* = 0.21).

## 4 Discussion

### 4.1 Overview

The purpose of this paper was to provide more detailed information on the SIT's reliability and validity to help support its potential usefulness. We conclude that the SIT does what it was designed to do: it provides a method to measure users' impressions of tailored health information in apps. Factor analyses were consistent with a single common factor, reliability is adequate, and the validity analyses are consistent with our hypotheses. SIT scores obtained immediately after the intervention were related to the pattern of change in the key outcomes of patient activation and chronic disease self-efficacy.

### 4.2 Factor structure

Although the bifactor model yielded the best statistical fit, the pattern of indices clearly supports the practical use of a single SIT composite score. The strong general factor accounted for a large portion of the common variance, while the specific factors contributed little unique and reliable information. This suggests that participants' responses primarily reflect an overarching perception that the information they received was well tailored to their needs, rather than distinct dimensions of tailoring. Accordingly, the SIT scale can be interpreted and scored as a unidimensional measure of perceived tailoring, simplifying its use in both research and applied settings while retaining conceptual coherence with the elaboration likelihood model's emphasis on personal relevance as the key mechanism of tailored communication.

Tailored health communications are more effective because they are perceived as more personally relevant (2). However, for a message to be perceived as relevant, it must first be cognitively accessible which is a concept known as “cognitive fit” (41). Within the Elaboration Likelihood Model (ELM), comprehension is a necessary prerequisite for message elaboration. Therefore, the SIT scale includes items assessing “ease of understanding” not as a measure of the user's skill, but as a measure of the intervention's success in adjusting content to the user's specific cognitive needs. This has been shown to increase message relevance and decrease cognitive load (42). While some items in the SIT explicitly address ease of understanding, our bifactor analysis confirms that these items load onto a robust general factor representing perceived tailoring. This suggests that for users, especially those with low health literacy, the ability to understand the information is inextricably linked to the feeling that the information was “successfully” personalized for them. By reducing the cognitive load required to process the information, the digital tailoring allows the user to focus on the personal utility of the advice.

Overall, the SIT Scale was framed using tailored health communication literature, where personalized message design is viewed as a multidimensional concept that uses person-level assessment to modify content, framing, and feedback so the information fits more appropriately to the person's needs and context (e.g., personalization). In this framework, tailoring appears to work through complementary pathways including strengthening cognitive preconditions for learning (e.g., attention, comprehension, perceived personal relevance) and the ability to impact behavioral determinants (e.g., confidence, skills, intentions) that drive self-management behavior (43, 44). Additionally, consistent with other studies tailoring can be produced through a segmentation, adaptation, and matching process (45). In this scenario, users are segmented based on variables (e.g., age, education, language, cultural belief, disease severity), and, as a result, the delivery of information (e.g., verbal, written, digital) is adapted. Finally, the matching process can be considered the crucial step that encompass how content and delivery are aligned with the user variables. However, data identify key limitations including segmentation rarely using technology related variables, user-interaction data to drive reminders, and a focus on only one type of segmentation variable (46). The SIT scale may not be able to determine which specific segmentation variables are predominantly impacting the user experience. However, the SIT scale may be positioned as a tool that can address whether the personalization is working (and why) and provide an objective score of the tailored content to determine if further improvements are needed.

As a result, the SIT scale was conceptually designed to capture these proximal, receiver-centered effects of tailoring. This includes identifying whether the information is perceived as relevant, useful, and actionable which are theorized mechanisms by which modifiable and tailored content produces changes in self-management outcomes (43, 47). This approach is consistent with data suggesting that tailored (including computer-tailored) delivery of information outperforms non-tailored comparisons. Therefore, the data appear to supports the importance of measuring perceived “fit” and actionability as important features of personalizing and tailoring content to the individual (1, 47). Finally, our finding that SIT scores are associated with related changes in activation and disease self-efficacy appears to align with health literacy frameworks and activation theory that emphasizes confidence, abilities, skills, and knowledge needed to interpret and act on personalized health information (12, 48, 49).

Given the theoretical framework, in order to better frame the SIT scale as a digital measure, it must be noted that eHealth personalization involves adapting not only the content but also the technology driven cues (e.g., interface, delivery, feedback) that impact understanding (46). For example, in the Klooster framework, users can be segmented into demographic/health characteristics and also on digital technology and interaction related variables (e.g., eHealth literacy, usage patterns) which can then be matched to design and delivery adaptations (45, 46). Accordingly, the SIT scale's “ease of understanding” items may be presented as an indicator of successful digital matching (content -interface- delivery cues) instead of a solely text-based construct. As a result, the endpoint is to determine if the adaptations achieved a perceived “fit” for the user in real-world app interactions. Also, consistent with Klooster et al. observations that many personalization efforts underuse technology related variables and user interaction data (e.g., reminders) (45, 46), the SIT scale may be a pragmatic outcome measure. Even though the SIT scale may not isolate which specific digital cues drive user perceptions, it can evaluate whether the implemented digital adaptations are experienced as relevant, useful, and actionable.

### 4.3 Measurement invariance and reliability

Test–retest reliability, measured by ICCs, was moderate. The Bland–Altman plot showed a small but significant average bias but individual variability. In practical terms, the group average was stable, although changes in a single person's score must be interpreted with care. SEM and SDC estimates provide useful guidance on interpreting changes in SIT scores.

### 4.4 Validity

The correlations of measured to represent similar and unrelated constructs were consistent with hypotheses. The SIT correlated significantly with participants' ratings of the app's usefulness and with the PAM and CDSE. These findings are consistent with the hypothesized relation as the underlying commonality is that both are about how people see their ability to act on health information. Correlations with unrelated measures were close to zero, supporting divergent validity.

### 4.5 Predictive validity

The latent growth curve models showed that people with higher baseline SIT scores made greater changes in activation and disease management self-efficacy over the course of the study. The SIT did not predict initial levels (intercepts); it only predicted the slope of improvement. These findings provide preliminary evidence suggesting that feeling the information was relevant and usable was associated with greater change later.

### 4.6 Limitations

There are a few caveats. The sample included persons 40 years and older with chronic illness and low health literacy, so results may not apply to other groups. Follow-up was only three months. CFA fit indices were not ideal, and ICCs were moderate, so the scale is better suited for group-level research than individual monitoring. Missing data also may have influenced some results.

We observed a substantial ceiling effect, with many participants responding at high levels. While the bifactor model confirmed that a strong general factor dominates the scale—supporting its use as an essentially unidimensional measure—the results must be interpreted within the context of this observed ceiling effect and restricted variability. These distributional characteristics likely contributed to the high RMSEA values and the absence of scalar invariance, suggesting that while the scale effectively captures group-level perceptions, it is not yet optimized for monitoring subtle individual changes over time. Furthermore, because the SIT scale successfully predicted the slope of change in patient activation and self-efficacy rather than initial status, it is best positioned as a process measure to verify the mechanism by which personalization influences behavioral outcomes.

Future refinements should focus on expanding the response range to mitigate the ceiling effect and validating the scale in diverse, higher-literacy populations to ensure its broader generalizability. The negatively skewed distributions of SIT items indicate that participants overwhelmingly perceived the intervention content as well tailored to their needs. While such skewness and kurtosis are expected in user-centered interventions, they may also signal ceiling effects that constrain variability and attenuate correlations with other constructs. Future refinements to the SIT scale might explore expanding the response range or incorporating items that better differentiate among individuals with uniformly positive perceptions. Nevertheless, the pattern of high endorsement supports the scale's face validity and aligns with the intervention's design to deliver highly personalized information. Although the bifactor analysis indicated strong general-factor dominance, some multidimensionality may still exist, and future research should examine whether distinct facets of perceived tailoring emerge in different populations or intervention contexts.

### 4.7 Implications and future directions

Based on digital personalization frameworks, the SIT scale may be viewed as a receiver-centered outcome measure of whether digital tailoring and adaptation (e.g., content, interface cues, delivery/feedback features) achieves a meaningful perceived “fit” for the user. The SIT scale provides an interpretable score to identify if the implemented digital adaptations are perceived as relevant, useful, and actionable. As a result, while it lacks the ability to isolate specific digital cues that drive fit, it offers a practical metric to guide iterative optimization of tailored digital health interventions in older adults with low health literacy. The SIT scale is thus primarily intended to serve as a process measure and a mediating variable rather than a primary clinical outcome. While it captures an “outcome” in terms of whether the tailoring process was successful in the user's mind, its greatest value lies in its ability to explain the mechanisms of behavior change within digital health interventions.

Even with its limitations, the SIT scale may provide researchers and program designers with a simple way to determine whether efforts to tailor health information to users are successful. It provides a means of assessing mechanisms of behavior change in digital health interventions. Future studies should examine how the scale performs in other populations, assess longer-term predictive validity, and explore the smaller factors suggested by the CFA results.

## Data Availability

Data will be supplied to interested researchers on request. Requests to access these datasets should be directed to ro71@nova.edu.
